# Flavonoids as Promising Natural Compounds for Combating Bacterial Infections

**DOI:** 10.3390/ijms26062455

**Published:** 2025-03-10

**Authors:** Ying Liu, Jiajia Zhu, Zhenyi Liu, Yan Zhi, Chen Mei, Hongjun Wang

**Affiliations:** 1 Institute of Animal Husbandry and Veterinary Medicine, Beijing Academy of Agriculture and Forestry Sciences, Beijing 100097, China; liuying@baafs.net.cn (Y.L.); liuzylynn@163.com (Z.L.); bjzhiyan@sina.com (Y.Z.); 2 Institute of Animal Husbandry and Veterinary, Hubei Academy of Agricultural Sciences, Wuhan 430064, China; xmszjj@hbaas.com

**Keywords:** antibiotic resistance, multidrug-resistant bacteria, flavonoids, natural antimicrobial compounds, bacterial infections

## Abstract

The increasing emergence and dissemination of multidrug-resistant (MDR) bacterial pathogens have intensified the need for new antibiotics and alternative therapeutic strategies. Flavonoids, a diverse group of bioactive natural compounds found in plants, have shown significant promise as antibacterial agents. Flavonoids inhibit bacterial growth through various mechanisms, including disruption of cell wall synthesis, prevention of biofilm formation, disruption of cell membrane integrity, and inhibition of bacterial efflux pumps. These actions not only reduce bacterial viability but also enhance the efficacy of conventional antibiotics, offering a potential solution to antibiotic resistance. However, challenges such as poor bioavailability limit their clinical application. Recent advances in nanotechnology-based drug delivery systems, chemical modifications, and formulation techniques have shown promise in improving flavonoid bioavailability and therapeutic efficacy. This review evaluates the antibacterial mechanisms of flavonoids, explores their potential synergistic effects with antibiotics, and highlights strategies to overcome bioavailability issues. Our findings underscore the importance of continued research on flavonoids as promising candidates for innovative antibacterial therapies aimed at combating MDR bacterial infections.

## 1. Introduction

Antibiotics have played a critical role in treating both human and animal infections. However, the emergence and rapid spread of multidrug-resistant bacteria pose a serious global public health threat [1,2,3,4]. Therefore, it is essential to develop antibacterial drugs with novel mechanisms of action. Compared to chemically synthesized drugs, natural antimicrobial compounds offer several advantages, including accessibility [5,6], structural diversity [7], potent bioactivity, and multiple modes of action [8]. Natural products provide a versatile foundation for drug discovery, particularly in the field of antibiotics. However, many of the compounds derived from soil microorganisms, as discovered using traditional methods like the Waksman platform, face challenges such as rediscovery of known compounds and low yields. This highlights the need to explore new sources of antimicrobial agents [9,10].

Plants, as the most abundant source of biodiversity on Earth, have historically played a pivotal role in drug discovery. Notable examples include plant-derived molecules like quinine and artemisinin, which have had significant impacts on malaria treatment [11]. Unlike mammals, plants lack advanced immune systems and have evolved unique chemical defense mechanisms to protect themselves against microbial pathogens [12]. Despite their rich chemical diversity, traditional herbal medicines have been largely overlooked since the golden age of antibiotic discovery. Plant-derived natural products exhibit a wide range of medicinal properties, often functioning as endogenous molecules involved in growth, development, and pest resistance. Examples include plant hormones such as gibberellins and abscisic acid and phytoalexins like pisatin and resveratrol [13,14]. These bioactive molecules are also invaluable sources for the development of novel therapeutic agents.

Among the various bioactive compounds found in medicinal plants, flavonoids stand out due to their wide-ranging applications in pharmaceuticals, nutrition, and agricultural chemistry [15,16,17]. Flavonoids are characterized by their polyphenolic structure, which includes a benzopyranone scaffold where two aromatic rings are connected by a three-carbon chain (C6-C3-C6 system) [18,19]. Recent research has increasingly emphasized the antibacterial potential of flavonoids [20,21]. Numerous studies have demonstrated the ability of flavonoids to inhibit MDR through a variety of mechanisms, including the inhibition of bacterial cell wall synthesis, prevention of bacterial biofilm formation, disruption of bacterial cell membrane integrity, and inhibition of key efflux pumps. These diverse modes of action highlight the potential of flavonoids as effective agents in addressing antibiotic resistance [22,23]. Additionally, flavonoids have shown the ability to reverse antibiotic resistance, thereby enhancing the efficacy of conventional antibiotics [24]. These findings underscore the potential of flavonoids as natural antimicrobial agents and open new avenues for the development of novel antibacterial therapies.

In summary, exploring the antimicrobial properties of plant-derived natural products, particularly flavonoids, is of great significance. This comprehensive review evaluates the antibacterial potential of flavonoids, elucidates their mechanisms of action, and investigates the possible synergistic effects when combined with conventional antibiotics. Furthermore, addressing the current research gaps and challenges is essential for fully harnessing the potential of flavonoids as promising antimicrobial agents. This review offers valuable insights to guide the development of innovative antibacterial strategies aimed at combating bacterial infections.

## 2. Plants as a Potential Source of Antimicrobial Agents

Since the golden era of antibiotics, the traditional approach of screening secondary metabolites from soil microorganisms for novel antibacterial drugs has encountered significant challenges [25]. Issues such as complicated purification processes, high redundancy of discovered compounds, and low yields have hindered progress in this field. Recently, researchers have shifted their focus to alternative sources for antibacterial compounds. Noteworthy discoveries include Lugdunin, an antibiotic secreted by bacteria in the human nasal cavity [26]; Darobactin, isolated from gut bacteria of nematodes [27]; and the identification of Teixobactin and Platensimycin from previously uncultured microbial sources [28,29,30]. These findings highlight the vast potential of untapped domains like the human microbiome, insects, marine environments, and terrestrial ecosystems in the search for novel antibacterial agents. Consequently, exploring unconventional sources for antibacterial compounds has emerged as a critical direction in contemporary antibiotic research.

**Figure 1:**
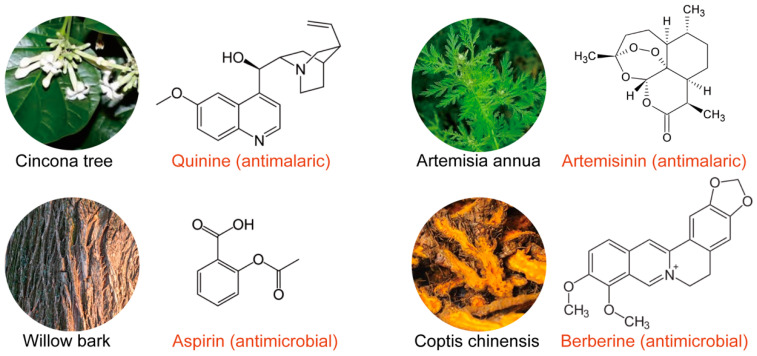
Bioactive compounds from medicinal plants. This figure highlights bioactive compounds derived from various medicinal plants with anti-malarial and antimicrobial properties. The *Cinchona* tree is a source of quinine (anti-malarial), *Artemisia annua* produces artemisinin (anti-malarial), willow bark provides aspirin (antimicrobial), and *Coptis chinensis* yields berberine (antimicrobial).

China, with its rich resources of traditional medicinal herbs, offers a natural advantage for the development and expansion of the herbal medicine industry. Advances in large-scale cultivation and modern extraction techniques have allowed the development of numerous natural medicines derived from numerous natural products, including artemisinin, berberine, and curcumin, among other herbal formulations [31]. Artemisinin, a highly effective anti-malarial compound, is extracted from *Artemisia annua*. In the 1970s, Chinese scientist Tu Youyou discovered its anti-malarial properties and spearheaded extensive research and development on its clinical applications [32,33,34]. Similarly, berberine, extracted from *Coptis chinensis*, is a potent antibacterial agent, while curcumin, derived from *Curcuma longa*, exhibits strong anti-inflammatory and antioxidant properties [35,36]. These compounds are applied not only in traditional medicine but also in the food and cosmetics industries, offering a wide range of applications, as illustrated in Figure 1.

In recent years, researchers have conducted in-depth studies on the chemical constituents of traditional herbs and identified many natural compounds with activity against MDR bacterial infections [37,38]. For instance, resveratrol, initially extracted from *Polygonum cuspidatum*, has since been identified in various other plants such as grape skins, red wine, peanuts, cocoa, and hawthorn [39,40]. Tea tree essential oil, originally used by Indigenous Australians to treat a variety of illnesses, is now commercially extracted through steam distillation of *Melaleuca alternifolia* leaves [41,42]. Moreover, tea polyphenols, secondary metabolites extracted from tea leaves, have demonstrated inhibitory effects on the growth and proliferation of various bacteria, including *Porphyromonas gingivalis*, *Mycobacterium tuberculosis*, *Staphylococcus aureus* (*S. aureus*), and *Pseudomonas aeruginosa* (*P. aeruginosa*) [43,44]. Thus, the untapped chemical diversity in traditional herbal medicines holds immense potential for the discovery of novel antibacterial compounds.

## 3. Overview of Flavonoids

### 3.1. Introduction and Classification of Flavonoids

**Figure 2:**
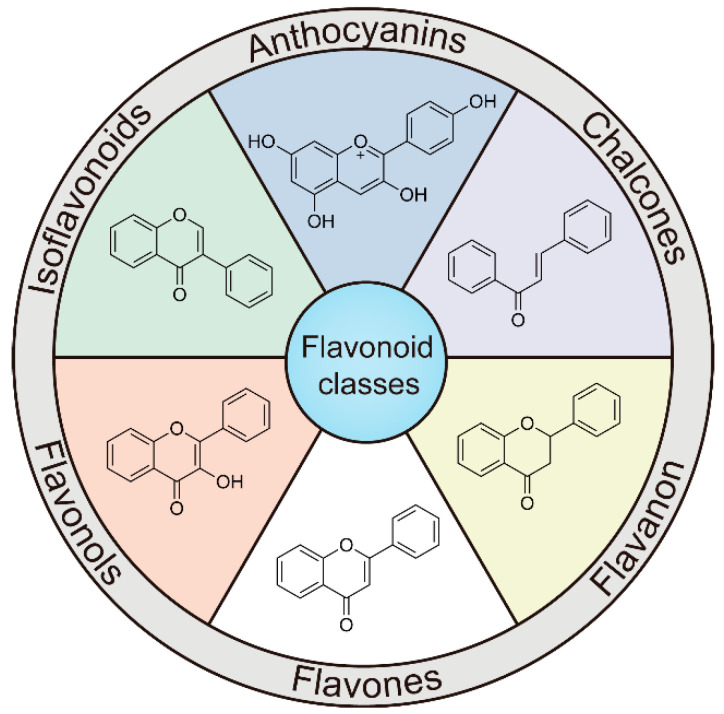
Chemical structure classifications of flavonoid classes. This diagram illustrates the primary classes of flavonoids, including anthocyanins, chalcones, flavanones, flavones, flavonols, and isoflavonoids.

Flavonoids are a diverse group of secondary metabolites widely distributed in the plant kingdom. Their molecular structure is characterized by a benzopyranone skeleton, with two aromatic rings connected by a three-carbon chain (C6-C3-C6 system) [18,19]. These structures can be further modified by the addition of various functional groups, such as hydroxyl, methoxy, and glycosyl groups, leading to the formation of a wide variety of flavonoid compounds [45]. Based on their degree of oxidation and different substituents, flavonoids are classified into several subclasses, including flavones, flavonols, flavanones, chalcones, and isoflavones, as shown in Figure 2 [46]. To date, over 5000 flavonoid compounds have been identified, many of which are natural products extracted from plants. For instance, the Echinacea species, known for its immune-boosting and anti-inflammatory effects, is a well-known natural source of flavonoids [47,48]. Additionally, some flavonoids, such as soy isoflavones and catechins, are synthesized chemically due to their broad biological activities and are widely applied in pharmaceutical and medical fields [49,50].

Plants naturally produce flavonoids as part of their defense mechanisms against oxidative stress, caused by free radicals and reactive oxygen species. These compounds exhibit strong antioxidant properties, enabling them to neutralize harmful substances within cells and prevent oxidative damage. Flavonoids also possess a broad spectrum of biological activities, including anti-inflammatory, antitumor, and antimicrobial effects, which help plants combat both biotic and abiotic stressors. Flavonoids are found in a variety of natural sources, including fruits (such as berries and citrus fruits), vegetables (such as onions and broccoli), legumes, tea, cocoa, and wine [51].

While the primary focus of flavonoid research has been on their antioxidant and anti-inflammatory properties, recent studies have also highlighted their potential in other therapeutic areas. Flavonoids, having evolved to adapt to diverse environmental conditions, are biodegradable, are low in toxicity, and are rapidly metabolized and excreted from the body. These characteristics make flavonoids promising candidates for further development in a wide range of applications, from pharmaceutical to environmental uses. Their safety profile and environmental friendliness underscore their potential as natural, sustainable therapeutic agents.

### 3.2. Biological Activities and Health Benefits Associated with Flavonoids

Flavonoids, with their diverse structures and widespread distribution in plants, offer a wide range of health benefits, primarily due to their potent antioxidant properties. These compounds play a key role in neutralizing free radicals, reducing oxidative stress, and preventing cellular damage, thereby contributing to the prevention of chronic diseases such as cardiovascular disease, cancer, and neurodegenerative disorders [52]. The health benefits of flavonoids are mediated through various mechanisms, including the modulation of cellular pathways, inhibition of inflammatory enzymes, and enhancement of immune responses [53].

In addition to their antioxidant and anti-inflammatory properties, flavonoids have shown promise in the prevention and treatment of cardiovascular diseases. Studies have demonstrated that flavonoid-rich diets are associated with improved endothelial function, reduced blood pressure, and decreased risk of atherosclerosis. Flavonoids such as quercetin and catechins have been found to modulate nitric oxide production, leading to vasodilation and improved blood flow, which are essential for cardiovascular health [54]. Furthermore, the anti-inflammatory properties of flavonoids contribute to their cardioprotective effects by reducing chronic inflammation, a key factor in the development of heart disease [55].

Flavonoids have also been studied for their potential in cancer prevention. Their ability to modulate cell proliferation, induce apoptosis, and inhibit angiogenesis makes them promising candidates for anticancer therapies [56,57]. Compounds such as epigallocatechin gallate (EGCG), commonly found in green tea, have demonstrated significant antitumor effects in various preclinical models. These flavonoids can disrupt key signaling pathways involved in cancer cell growth and metastasis, including the PI3K/Akt and MAPK pathways. Additionally, flavonoids may play a neuroprotective role by mitigating oxidative stress and inflammation, which are implicated in the pathogenesis of neurodegenerative diseases such as Alzheimer’s and Parkinson’s disease. Some flavonoids, like resveratrol, have shown potential in improving cognitive function and reducing amyloid plaque formation in animal models of Alzheimer’s disease [58,59].

Overall, the diverse biological activities of flavonoids, including antioxidant, anti-inflammatory, cardioprotective, anticancer, and neuroprotective effects, highlight their significant health benefits. These properties make flavonoids promising candidates for the development of novel therapeutic agents aimed at preventing and treating various chronic diseases.

## 4. Antibacterial Potential of Flavonoids

### 4.1. Antibacterial Activity of Flavonoids Against Various Bacterial Strains

Flavonoids, commonly found in plant-based herbal medicines, exhibit a broad spectrum of antimicrobial activity, making them promising candidates for the development of new antibacterial agents. Researchers have identified 459 plant-derived antimicrobial compounds, with approximately half being phenolic derivatives, primarily flavonoids [52,60]. These compounds effectively inhibit the growth and reproduction of various bacterial strains, including drug-resistant pathogens. Notable examples include glabrol and artocarpin, which have demonstrated significant activity against drug-resistant bacteria. Furthermore, certain flavonoid compounds, such as glabrol and kuwanon H, exhibit antibiotic-enhancing effects and have been shown to potentiate the antibacterial activity of specific antibiotics [39,42,52,53]. In addition to their potent antimicrobial effects, flavonoids have evolved over time to demonstrate high environmental and biological adaptability. They are rapidly metabolized and excreted from the body, preventing accumulation and minimizing environmental impact. Moreover, flavonoids exhibit good biodegradability and low toxicity, characteristics that make them promising candidates for the development of environmentally friendly and safe antimicrobial drugs.

Flavonoids also possess significant antimicrobial properties against various microbial pathogens. For instance, flavonoids have exhibited potent antibacterial effects against resistant bacterial strains, including Methicillin-resistant *Staphylococcus aureus* (MRSA) and vancomycin-resistant Enterococci (VRE), even restoring sensitivity to colistin, a last-resort antibiotic [61]. Furthermore, flavonoids have shown significant inhibitory effects against fungal infections caused by Candida albicans and certain molds [62]. Overall, the antimicrobial activity of flavonoids underscores their potential as natural agents for the development of novel antimicrobial therapies.

However, it is important to note that the safety and cytotoxicity of certain flavonoids require further investigation. Some studies have reported significant cytotoxic effects associated with specific antimicrobial flavonoids, such as α-mangostin, highlighting the need for structural optimization to improve their safety profile [63]. Additionally, determining the appropriate and safe dosage of flavonoids in food and food additives remains a key area of research. Despite these challenges, the consumption of foods rich in flavonoids continues to be associated with notable health benefits. Continued research into the mechanisms of action and optimization of flavonoid-based interventions holds great promise for the development of novel therapeutic strategies in the fields of medicine and public health.

### 4.2. Potential Synergistic Combinations of Flavonoids with Antibiotics or Other Natural Compounds

The exploration of synergistic combinations involving flavonoids, antibiotics, and other natural compounds holds significant potential for combating bacterial infections. Researchers have been investigating how flavonoids may enhance the antibacterial activity of antibiotics. Studies have demonstrated that the combination of flavonoids with antibiotics such as tetracycline, erythromycin, and ciprofloxacin can result in enhanced efficacy compared to individual treatments [64]. This synergistic effect may allow for lower antibiotic doses, reducing the risk of antibiotic resistance development. In addition to antibiotics, synergistic interactions between flavonoids and other natural compounds, such as plant extracts or essential oils, have also been explored. Combining flavonoids with these natural agents can lead to enhanced antibacterial activity, potentially through complementary mechanisms. Such synergistic combinations offer promising avenues for developing novel antimicrobial formulations that leverage the benefits of multiple natural compounds.

The mechanisms underlying these synergistic effects include the following: (a) Enhanced Penetration: Flavonoids can increase the permeability of bacterial cell membranes, improving the entry of antibiotics or other antimicrobial agents into bacterial cells, thereby enhancing antibacterial efficacy. Flavonoids such as quercetin and catechins have been shown to disrupt bacterial membrane integrity by interacting with membrane lipids, leading to increased membrane fluidity and permeability [65]. This disruption facilitates greater penetration of antibiotics into bacterial cells. For instance, EGCG can bind to lipid bilayers, causing membrane depolarization and increased permeability, which enhances the uptake of antibiotics like oxacillin in MRSA [66]. Additionally, flavonoids may alter the fatty acid composition of bacterial membranes, further affecting membrane properties and antibiotic susceptibility [67]; (b) Efflux Pump Inhibition: Many bacteria use efflux pumps to expel antibiotics, reducing their intracellular concentration. Flavonoids have been shown to inhibit these efflux pumps, allowing antibiotics to accumulate inside bacterial cells and increasing their effectiveness. For example, baicalein has been reported to inhibit the NorA efflux pump in *S. aureus*, enhancing the intracellular concentration of antibiotics like ciprofloxacin [68]; (c) Target Site Modification: Flavonoids can interact with bacterial enzymes or proteins, modifying their structure or activity. This interaction may enhance the binding or action of antibiotics at their target sites, leading to increased antibacterial activity; (d) Modulation of Bacterial Resistance Mechanisms: Flavonoids may interfere with bacterial resistance strategies, such as biofilm formation or quorum sensing, making bacteria more vulnerable to antibiotics. This modulation can improve the efficacy of antibiotics in eradicating infections [69,70,71,72]. Furthermore, flavonoids can affect membrane-associated proteins, altering membrane potential and disrupting energy-dependent processes essential for bacterial survival. By compromising membrane integrity and function, flavonoids make bacterial cells more susceptible to antibiotics. This combined action weakens bacterial defense mechanisms and allows antibiotics to exert their effects more efficiently [73,74].

The implications of these synergistic combinations are significant for combating bacterial infections. Synergy between flavonoids and antibiotics allows for lower antibiotic dosages, reducing selective pressure for the development of antibiotic resistance. Moreover, these combinations may help overcome drug-resistant bacteria by targeting multiple bacterial pathways simultaneously. Synergistic approaches using flavonoids and other natural compounds offer alternative strategies for the treatment of bacterial infections and could provide solutions to the growing problem of antibiotic resistance.

### 4.3. Antibacterial Efficacy of Flavonoids in In Vivo Studies

Recent in vivo studies have reinforced the antibacterial potential of flavonoids, highlighting their therapeutic applicability within biological systems. Flavonoids such as quercetin, diosmin, and baicalein have demonstrated significant efficacy against bacterial infections in various animal models. Quercetin treatment in mice infected with *S. aureus* resulted in a substantial reduction of bacterial loads in organs like the liver and spleen, enhanced survival rates, and modulation of the immune response through decreased pro-inflammatory cytokines [75]. Similarly, citrus-derived flavonoids—diosmin, myricetin, and neohesperidin—exhibited potent antivirulence properties against *P. aeruginosa* in a zebrafish embryo model by reducing biofilm formation, downregulating virulence genes, and effectively lowering mortality without embryotoxicity [76]. Moreover, baicalein and *Anethum graveolens* L. fruit extracts have shown therapeutic effects in mice with *E. coli*-induced bacterial colonic diseases, decreasing intestinal bacterial counts and improving intestinal tissue health. This suggests a complex mechanism involving modulation of gut microbiota and reinforcement of intestinal barrier function [77,78].

Further research has explored the impact of flavonoids on various pathogens. Specific flavonoids have been found to significantly lower gastric bacterial loads of *Helicobacter pylori* in mice, easing gastritis symptoms. This effect is achieved through the inhibition of urease activity, a critical enzyme that enables the bacterium to survive in acidic conditions [79]. While animal studies are prevalent, clinical data are limited. Some studies suggest that consumption of flavonoid-rich foods, such as cranberry juice containing proanthocyanidins, may reduce the incidence of urinary tract infections by preventing bacterial adhesion [80]. Additionally, flavonoid-rich diets have also been associated with improved outcomes in patients with chronic infections; however, more rigorous clinical trials are necessary to confirm these findings.

These in vivo studies highlight the efficacy of flavonoids in combating bacterial infections within living organisms. They demonstrate the ability of flavonoids to reduce bacterial loads, inhibit virulence factors, modulate immune responses, and enhance host outcomes without notable toxicity. These findings are essential for bridging the gap between laboratory research and clinical applications, underscoring the importance of further in vivo and clinical studies to fully explore their potential as antibacterial agents.

### 4.4. Bioavailability and Strategies to Enhance the Efficacy of Flavonoids

Despite their promising antibacterial properties, the clinical application of flavonoids is often limited due to poor bioavailability. Factors contributing to low bioavailability include poor solubility, instability in the gastrointestinal tract, rapid metabolism, and low absorption rates [81]. Flavonoids are extensively metabolized in the liver and intestines, leading to the formation of various metabolites that may have reduced biological activity [82,83].

To overcome these limitations, several strategies have been explored to enhance the bioavailability and therapeutic efficacy of flavonoids. One approach involves the use of advanced drug delivery systems. Nanotechnology-based carriers like nanoparticles, liposomes, and nanoemulsions have been developed to improve the solubility, stability, and absorption of flavonoids. For example, encapsulating flavonoids in nanoparticles can protect them from degradation, enhance their cellular uptake, and provide controlled release [84]. For instance, quercetin-loaded chitosan nanoparticles have shown increased antibacterial activity against *S. aureus* compared to free quercetin due to improved bioavailability [85]. Liposomes, phospholipid-based vesicles, can encapsulate flavonoids, enhancing their absorption and targeting infection sites more effectively [86].

Chemical modifications of flavonoids represent another strategy to improve their bioavailability. Structural modifications, such as methylation, glycosylation, or acylation, can enhance lipophilicity and stability, improving membrane permeability and absorption [87]. Methylated flavonoids, such as 7-*O*-methylapigenin and 7-*O*-methylnaringenin, have shown increased metabolic stability and enhanced antibacterial activity [88,89]. Prodrug approaches, where flavonoids are chemically modified to improve pharmacokinetic properties and then converted back to the active form in the body, have also been investigated to enhance efficacy [90]. Additionally, the use of absorption enhancers is another promising method to improve flavonoid bioavailability. Co-administration with substances that inhibit metabolic enzymes or efflux transporters can increase absorption. For instance, piperine, an alkaloid from black pepper, has been shown to enhance the bioavailability of curcumin by inhibiting its metabolism [91]. Formulation techniques such as solid dispersion, inclusion complexes with cyclodextrins, and microencapsulation have also been employed to improve the solubility and stability of flavonoids, thereby enhancing their absorption [92,93]. Cyclodextrin inclusion complexes of baicalein have demonstrated improved solubility and antibacterial activity, illustrating the effectiveness of these techniques [94].

Enhancing the bioavailability of flavonoids is crucial for translating their in vitro efficacy into clinical success. Continued research into advanced drug delivery systems, chemical modifications, and formulation techniques holds promise for optimizing flavonoid-based antibacterial therapies. By addressing bioavailability challenges, these strategies can facilitate the development of effective flavonoid formulations for clinical use.

## 5. Antibacterial Mechanism of Flavonoids

### 5.1. Inhibition of Bacterial Cell Wall Synthesis

Flavonoids have been shown to inhibit bacterial growth by interfering with the synthesis of the cell wall, a critical component for bacterial survival and structural integrity. The bacterial cell wall, primarily composed of peptidoglycan, plays an essential role in maintaining cell shape and protecting against osmotic pressure. Inhibition of cell wall synthesis leads to cell lysis and ultimately bacterial death [95].

One of the mechanisms by which flavonoids inhibit cell wall synthesis is through the inhibition of key enzymes involved in peptidoglycan biosynthesis. Flavonoids can target enzymes such as peptidoglycan glycosyltransferases, which are essential for the polymerization and cross-linking of peptidoglycan strands [96]. For example, quercetin has been demonstrated to inhibit bacterial enzymes like MurB, crucial in the early stages of peptidoglycan biosynthesis. This inhibition disrupts cell wall formation, leading to weakened cell structure and eventual cell lysis [97]. Additionally, flavonoids have been reported to interfere with the synthesis of UDP-N-acetylglucosamine, a key precursor in the peptidoglycan biosynthesis pathway [98,99], hindering the proper assembly of peptidoglycan and further compromising cell wall integrity.

Specific flavonoids exhibit selective inhibition of bacterial cell wall synthesis. Flavonoids in Miang extract, particularly pyrogallol, have been shown to inhibit enzymes such as MurG, MurC, and MraY, which are essential for the production of lipid II—a critical precursor in peptidoglycan construction [100]. By targeting this initial step, Miang extract effectively prevents the formation of essential cell wall components, leading to growth inhibition and bacterial death [101]. Similarly, baicalein, a flavonoid derived from *Scutellaria baicalensis*, has demonstrated strong inhibitory effects on bacterial cell wall synthesis by disrupting the function of autolysins, enzymes involved in cell wall remodeling and turnover [102].

The inhibition of cell wall synthesis by flavonoids holds particular promise for combating drug-resistant bacteria. Bacterial resistance mechanisms, such as the production of β-lactamase enzymes, often render β-lactam antibiotics ineffective. Flavonoids, however, can target non-β-lactam pathways in cell wall synthesis, allowing them to remain effective against β-lactam-resistant strains. This provides an alternative or complementary therapeutic approach to conventional antibiotics. Additionally, flavonoids’ ability to modulate cell wall integrity makes them promising candidates for combination therapies, potentially enhancing the efficacy of existing antibiotics [64].

### 5.2. Inhibition of Bacterial Biofilm Formation

Flavonoids have shown promise in disrupting bacterial biofilm formation, a critical factor in the persistence of infections caused by MDR pathogens. Biofilms are structured communities of bacteria encased in a self-produced extracellular matrix, which protects them from both the host immune system and antimicrobial agents [103]. The ability of flavonoids to inhibit biofilm formation offers a potential strategy to enhance the efficacy of conventional antibiotics.

Research has demonstrated that flavonoids can interfere with the initial stages of biofilm formation by inhibiting bacterial adhesion to surfaces. For instance, flavonoids have been shown to reduce the adhesion of various bacterial strains, including *S. aureus*, *P. aeruginosa*, and *Candida* spp., to medical devices and host tissues. This reduction in adhesion can significantly hinder biofilm establishment [104]. Additionally, flavonoids may disrupt the established biofilm architecture. Certain flavonoids have been reported to enhance the susceptibility of biofilm-embedded bacteria to antibiotics, facilitating their eradication [105]. This is particularly relevant for treating chronic infections, where biofilms pose a significant challenge. The mechanisms through which flavonoids exert their anti-biofilm effects include the modulation of signaling pathways involved in biofilm development, such as quorum sensing [106]. By interfering with these signaling mechanisms, flavonoids can inhibit the expression of genes necessary for biofilm maturation and stability.

In conclusion, the ability of flavonoids to inhibit bacterial biofilm formation not only underscores their potential as antimicrobial agents but also highlights their role in enhancing the efficacy of existing treatments against biofilm-associated infections.

### 5.3. Disruption of Bacterial Cell Membrane Integrity

Flavonoid compounds possess the ability to disrupt bacterial cell membranes by integrating into the membrane structure and interfering with its function, ultimately leading to cell death [107]. These compounds contain both hydrophobic aromatic rings and hydrophilic functional groups, such as hydroxyl or methoxy groups, which enable them to interact with the lipid bilayer. This interaction compromises membrane integrity and disrupts its functionality, leading to bacterial cell death [108,109,110,111]. Additionally, flavonoids can interact with various lipid molecules present in the bacterial cell membrane, causing changes in membrane structure and fluidity that further contribute to membrane disruption and loss of function. For instance, resveratrol can perturb the fluidity and stability of bacterial membranes by interacting with phospholipid molecules, thereby impairing normal membrane function [40,112].

Flavonoids can also affect the proton motive force (PMF) across bacterial membranes, which is essential for energy production and nutrient transport. Kuwanon G, a flavonoid found in Morus species, exhibits significant antimicrobial activity against MRSA by targeting the PMF and disrupting membrane permeability [38]. Similarly, isorhamnetin has been shown to interfere with the PMF, leading to the disruption of membrane function and subsequent elimination of MRSA. Additionally, flavonoids such as α-mangostin and isobavachalcone specifically target the phospholipid bilayer of bacterial membranes, inducing significant alterations in membrane structure and fluidity. By dissipating the bacterial PMF and causing corresponding changes in intracellular ATP levels, these compounds lead to bacterial cell death [61]. The membrane-targeting properties of flavonoids represent a promising avenue for the development of novel antimicrobial agents. Their ability to disrupt membrane integrity and interfere with essential membrane functions makes them effective against a broad spectrum of bacteria, including drug-resistant strains. This mechanism of action is particularly valuable because it reduces the likelihood of cross-resistance with conventional antibiotics that target other cellular processes.

### 5.4. Flavonoids as Inhibitors of Bacterial Efflux Pumps

Efflux pumps play a crucial role in bacterial resistance by actively expelling antibiotics and other antimicrobial agents out of the cell, thereby reducing their intracellular concentrations and effectiveness [113,114]. Many MDR bacteria utilize efflux pumps as a primary defense mechanism, making the inhibition of these pumps a promising strategy to restore antibiotic efficacy. Flavonoids have been studied for their ability to inhibit bacterial efflux pumps, enhancing the intracellular retention of antibiotics and other antimicrobial agents [115].

Flavonoids are known to inhibit various types of bacterial efflux pumps, such as those from the resistance-nodulation-division (RND) and major facilitator superfamily (MFS) families. By interfering with the activity of these pumps, flavonoids can prevent the expulsion of antibiotics from bacterial cells, thereby increasing their intracellular concentration and restoring their antibacterial activity [116]. For instance, EGCG, a major flavonoid found in green tea, has been shown to inhibit the activity of the NorA efflux pump in MRSA, leading to increased intracellular accumulation of antibiotics like ciprofloxacin [68]. This inhibition sensitizes *S. aureus* to antibiotics that it would otherwise expel, reducing the bacteria’s resistance to treatment.

Several flavonoids have demonstrated the ability to inhibit efflux pumps in both Gram-positive and Gram-negative bacteria. Quercetin, for example, has been shown to block the AcrAB-TolC efflux pump in *E. coli*, one of the most prominent RND family efflux systems responsible for exporting a wide range of antibiotics [117]. Inhibition of this pump increases the intracellular concentration of antibiotics, making *E. coli* more susceptible to drugs such as tetracycline and chloramphenicol. Similarly, kaempferol has been reported to inhibit the MexAB-OprM efflux system in *P. aeruginosa*, another notorious MDR pathogen, enhancing the efficacy of antibiotics that are typically expelled by this pump [118].

The ability of flavonoids to inhibit bacterial efflux pumps holds significant clinical promise, particularly in the treatment of infections caused by MDR bacteria. By inhibiting efflux pumps, flavonoids can lower the minimum inhibitory concentration of antibiotics, potentially reducing the required dosage and minimizing side effects. Additionally, combining flavonoids with conventional antibiotics may provide a synergistic effect, improving treatment outcomes against resistant bacterial strains [119]. This combination approach could offer an alternative strategy to combat the growing issue of antibiotic resistance in both hospital and community settings.

**Figure 3:**
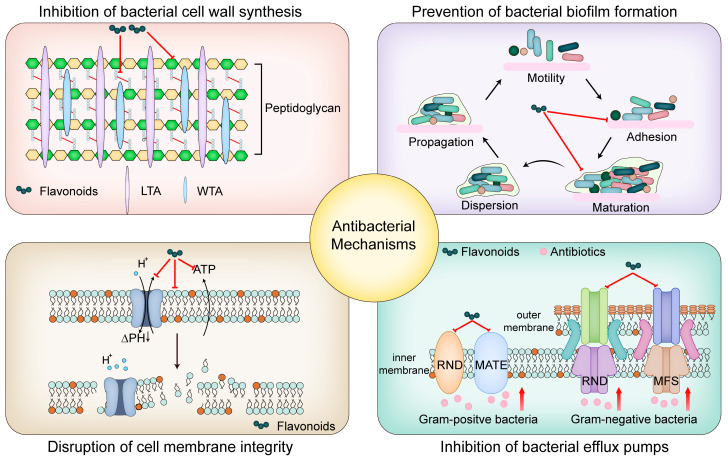
Antibacterial mechanism of flavonoids. This figure illustrates the antibacterial actions of flavonoids, including inhibition of bacterial cell wall synthesis, prevention of biofilm formation, disruption of cell membrane integrity, and inhibition of bacterial efflux pumps, which enhance the effectiveness of antibiotics against bacterial infections.

In summary, flavonoids exhibit significant antibacterial activity through diverse mechanisms, including inhibition of cell wall synthesis, disruption of biofilm formation, impairment of cell membrane integrity, and inhibition of bacterial efflux pumps (Figure 3 and Table 1). These multifaceted actions not only underscore the potential of flavonoids as effective antibacterial agents but also highlight their promise in overcoming current challenges associated with antibiotic resistance. Building on these mechanistic insights, it is essential to explore the practical applications of flavonoids in clinical settings and identify areas where further research is needed.

## 6. Conclusions

Flavonoids exhibit significant antibacterial potential against a wide range of bacterial pathogens, including MDR strains. Their diverse mechanisms of action—such as inhibiting cell wall synthesis, disrupting biofilm formation, impairing membrane integrity, and inhibiting efflux pumps—make them promising candidates for developing new antimicrobial therapies. Importantly, recent in vivo studies have validated these effects within animal models, demonstrating the efficacy of flavonoids like quercetin, diosmin, and baicalein in reducing bacterial infections and improving host outcomes without notable toxicity. The inclusion of clinical observations further suggests potential applications in human health. The synergistic effects observed when flavonoids are combined with conventional antibiotics or other natural compounds enhance their therapeutic potential.

However, despite these promising findings, several challenges remain in translating flavonoids into clinical applications. One major hurdle is the poor bioavailability of flavonoids due to their low solubility, rapid metabolism, and limited absorption. Future research should focus on developing advanced drug delivery systems, such as nanoparticles, liposomes, and inclusion complexes, to enhance their bioavailability and therapeutic efficacy. Additionally, chemical modifications and prodrug strategies could be explored to improve their pharmacokinetic properties.

Comprehensive clinical trials are necessary to evaluate the safety, optimal dosing, and efficacy of flavonoids in humans. Investigating the long-term effects and potential toxicity is crucial for their development as therapeutic agents. Further studies should also delve into the molecular mechanisms underlying their antibacterial activity, which could lead to the identification of more potent derivatives and the optimization of their use in combination therapies. Moreover, exploring the synergistic interactions between flavonoids and existing antibiotics can provide valuable insights into overcoming antibiotic resistance. Understanding how flavonoids modulate bacterial membranes and inhibit resistance mechanisms may lead to novel treatment strategies. Research into the effects of flavonoid combinations with other natural compounds could also uncover new avenues for antimicrobial therapy.

In conclusion, by addressing these challenges through targeted research efforts, flavonoids have the potential to become a crucial component in the fight against bacterial infections and antibiotic resistance. Their natural abundance, diverse mechanisms of action, and ability to enhance the efficacy of existing antibiotics make them promising candidates for future antimicrobial drug development.

## Figures and Tables

**Table 1:** Antibacterial mechanisms and sources of natural plant flavonoids.

Flavonoid Classes	General Structure of Flavonoid	Example	Natural Sources	Strains	MIC (μg/mL)	Target	References
Anthocyanins	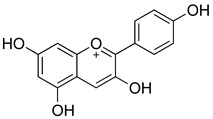	Cyanidin-3-O-glucoside	Berries (e.g., blueberries, blackberries, raspberries), red cabbage, cherries, purple corn	*S. aureus*, *E. coli*, *S. typhimurium*, *L. monocytogenes*	64–512	Disruption of bacterial cell membrane integrity Inhibition of biofilm formation	[120]
Chalcones	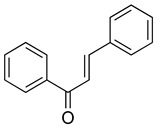	Licochalcone A	Licorice root (Glycyrrhiza uralensis)	*S. aureus*, MRSA, *B. subtilis*, *E. coli*	2–64	Disruption of bacterial cell membrane integrity Inhibition of fatty acid synthesis Inhibition of bacterial DNA gyrase	[121,122]
		Isobavachalcone	Dorstenia barteri	*S. aureus*, MRSA, *E. faecium*	1–8	Disruption of bacterial cell membrane structure and function	[61]
Flavanones	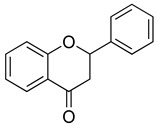	Naringenin	Citrus fruits (e.g., grapefruits, oranges), tomatoes	*B. subtilis*, *P. aeruginosa*	50–200	Disruption of bacterial cell membrane integrity Inhibition of bacterial quorum sensing Interference with nucleic acid synthesis	[123,124]
Flavones	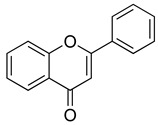	Apigenin	Parsley, celery, chamomile, thyme	*S. aureus*, *B. subtilis*, *E. coli*, *L. monocytogenes*	25–100	Disruption of bacterial cell membrane integrity Inhibition of nucleic acid synthesis	[125]
Flavonols	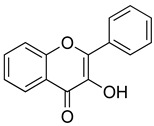	Quercetin	Tea, broccoli, kale, beans, spinach	*S. aureus*, *P. aeruginosa*	8–256	Disruption of bacterial cell walls and cell membrane Reduction of expression of virulence factors	[44]
		Kaempferol	Kale, beans, tea, spinach, broccoli	*S. aureus*, *E. coli*, *K. pneumoniae*, *P. aeruginosa*	0.5–625	Disruption of bacterial cell membranes Inhibition of fatty acid biosynthesis and biofilm formation Suppression of DNA gyrase and helicase activity	[126]
Isoflavonoids	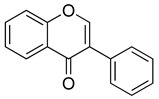	Genistein	Soybeans, soy products, legumes (e.g., chickpeas, lentils)	*S. aureus*, *A. hydrophila*	32–256	Inhibition of bacterial DNA gyrase and topoisomerase IV Disruption of cell membrane integrity Inhibition of protein synthesis	[127,128]
